# Correction: Navigating board dynamics: Configuration analysis of corporate governance’s factors and their impact on bank performance

**DOI:** 10.1371/journal.pone.0319614

**Published:** 2025-02-19

**Authors:** Safdar Husain Tahir, Sadeen Ghafoor, Muhammad Zulfiqar, Mushtaq A. Sajid, Huma Illyas

There is an error in affiliation 3 for author Muhammad Zulfiqar. The correct affiliation 3 is: Zhejiang Gongshang University Hangzhou College of Commerce, Hangzhou, People's Republic of China.
